# Current Research of Phytochemical, Medicinal and Non-Medicinal Uses of *Uncaria gambir* Roxb.: A Review

**DOI:** 10.3390/molecules27196551

**Published:** 2022-10-03

**Authors:** Indah Putri Munggari, Dikdik Kurnia, Yusi Deawati, Euis Julaeha

**Affiliations:** Department of Chemistry, Faculty of Mathematics and Sciences, Universitas Padjadjaran, Jl. Raya Bandung-Sumedang Km. 21 Jatinangor, Sumedang 45363, Indonesia

**Keywords:** *Uncaria gambir* Roxb., catechin, flavonoids, alkaloids, antioxidant

## Abstract

*Uncaria gambir* Roxb. is a plant from Southeast Asia and is widely used as an alternative medicine with various applications. This plant has been widely used in traditional medicine. This paper aims to provide information on *U. gambir*, a summary of data on phytochemicals and on medical and nonmedical activities. Phytochemical studies reveal biologically active constituents such as flavonoids, phenolics, and alkaloids. Various studies have shown that extracts and compounds obtained from *U. gambir* have medical uses for their antioxidant, antibacterial, anti-helminthic, anticancer, antifungal, anti-inflammatory, anti-hyperglycemic, anti-hyperuricemic, anti-lipid peroxidation, antihyperlipidemic and other properties. In addition, this extract has other uses, such as adsorbent for dyes and metal ions, as well as corrosion inhibition. Thus, *U. gambir*, which is commonly used in traditional medicine, is a potential plant for many therapeutic applications and prospects for drug development as well as other applications such as adsorbent and corrosion inhibition.

## 1. Introduction

The use of traditional medicine, especially from plants, has received much attention for alternative medicine apart from modern medicine around the world [1,2,3,4]. As sources of human medicine, natural products from medicinal plants offer a wide range of biological activities and skeletal structures. Most plants contain secondary metabolites with biological characteristics that provide opportunities for obtaining medicines for the treatment of diseases directly from natural resources [5]. *Uncaria gambir* Roxb. is a plant belonging to the Rubiaceae family. This plant comes from Southeast Asia, especially Indonesia and Malaysia [6,7]. Gambir plants usually grow at an altitude between 200 and 800 m above sea level [8]. In Indonesia, gambir was a trading commodity starting in the early 19th century [9]. Currently, Indonesia is a supplier of 80% of gambir commodities in the world market [10].

The gambir plant is often used as traditional medicine and is prescribed to relieve minor ailments such as fever, diarrhea, headache, runny nose, cough, and stomach ache [11]. In addition, gambir has also been used by communities for the treatment of wounds, ulcers, asthma, headaches, gastrointestinal diseases, bacterial/fungal infections, gums, tooth pain, cancer, cirrhosis, diabetes, rheumatism, dysentery, and urinary tract inflammation [12]. The benefits of gambir could derive from the activity of the compounds contained. Therefore, it is important to know the content of compounds in gambir and the activity it causes. The purpose of writing this review is to provide information related to pharmacological activity and the content of compounds in gambir.

In 1968, Carrick et al. [13] conducted an extensive review of the chemical composition of Malayan plants and reported on the phytochemistry of gambir. They identified that gambir contains alkaloid compounds. With the continuous improvements in medicinal plant research methods, increasing numbers of compounds including alkaloids, flavonoids, and tannins have been discovered from gambir. Modern studies have shown that gambir has pharmacological activities such as anticancer [14], enzyme inhibitory [15], and hypoglycemic [16] activity due to flavonoid compounds [17].

As described in detail in previous studies, gambir provides diverse biological functions. Gambir is considered an excellent source of antioxidant compounds. Recent research on gambir has involved other than medical applications, for example, as an adsorbent and a corrosion-inhibition agent. Because of the extensive investigation of *Uncaria gambir* Roxb. in the last ten years, a wide variety of biological as well as non-biological activities are described in this review. In this review, we mainly summarize the reports related to gambir over the last ten years, in which a wider field of research has been reported.

This review focuses on the complete phytochemical information including medical and nonmedical uses of gambir. The purpose of this review is to facilitate the further study of gambir through a systematic summary of flavonoids and alkaloids, simultaneously exploring the potential of gambir in the medical field and discovering its potential in other fields. This review ends with conclusions and perspectives that can stimulate further development of the usefulness of *Uncaria gambir* Roxb. Although several review articles of the same scope have been published [18,19], a thorough explanation has not been reported. Other reviews focus on different and broader aspects such as the phytochemistry and pharmacology of the *Uncaria genus* [20] and the pharmacology and structure of alkaloid compounds of the *Uncaria genus* [21].

## 2. Ethnobotany of *Uncaria gambir* Roxb.

**Figure 1:**
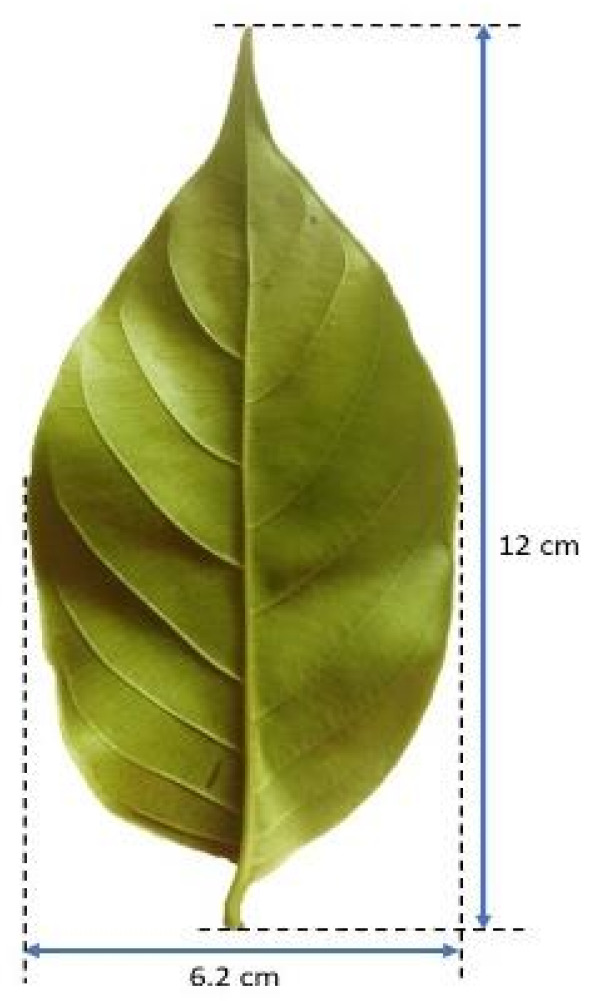
Macroscopic characteristics of *Uncaria gambir* leaf.

Gambir plants can grow to a height of about 2.4 m, with leaves about 8–14 cm, with a width of about 4.0–7.5 cm (Figure 1) [22,23]. The flowers are tubular and hairy with round heads about 6–8 cm. The fruit is nearly cylindrical and has a length of less than 2 cm [24]. Gambir plants can only grow under certain conditions in all types of soil with a pH range of 4.8–5.5. Plants need to be planted at an altitude of 200–800 m above sea level, with high rainfall throughout the year that is >200 mm/month or a total annual rainfall of 3000–4000 mm. In addition, an air temperature of 20–36 °C is required with a humidity of around 70–85% and a land slope of 15% [25].

Gambir plants, like most other plantation crops, can be propagated vegetatively and generatively. Propagation through the method of cuttings (vegetative) at the location of the mother tree showed low success, namely, 15–40% [26]. In addition, this method is difficult to transport, easily damaged, and sensitive to drought. Another vegetative method, namely layering, can achieve a higher success rate of 80%. This method has the disadvantage that it is difficult to transfer from the parent tree because few roots are formed [27,28].

## 3. Phytochemical of *Uncaria gambir* Roxb.

Gambir was first reported in the early 1900s [29]. The results of phytochemical screening showed that gambir contains secondary metabolites such as flavonoids, alkaloids, tannins, terpenoids, and saponins [30,31].

### 3.1. Flavonoids

The flavonoid biosynthetic pathway in *Uncaria gambir* was studied by Das and Griffiths in 1967 [32]. This study is in accordance with the previous hypothesis by Birch andDonovan [33] that ring A of flavonoids is formed by the condensation of three acetyl units, while ring B is derived from phenyl. According to Das and Griffiths, the administration of ^14^CO_2_ and [1-^14^C]-acetate showed the formation of (+)-[^14^C]-catechins in gambir. It was later proven by alkaline degradation that the administration of ^14^CO_2_ resulted in the same labeling of rings A and B in catechins to phloroglucinol and protocatechuic acid, whereas [1-^14^C]-acetate gave rise to labeling mostly in ring A. Then, in 1974, Das revealed that (+)-catechin and (-)-epicatechin were the main flavonoids present together with small amounts of three unidentified polyphenols [34].

Then a few years later, it was discovered that catechins (**1**) were the most common flavonoid compounds found in gambir [35]. A catechin compound is reported to be the main bioactive compound in gambir [36]. Anggraini et al. [37] isolated flavonoid compounds, namely, catechins and epicatechins. This gambir sample was extracted using hot water for 1.5 h, then separated and analyzed using HPLC. In 2003, the content of catechins in gambir was analyzed from aqueous extract using several extraction methods and three analytical methods: hot plate, ultrasonic batch, and shaker. The results show that the extraction method using a hot plate with water extract at a temperature of 52–75 °C had the highest catechin content, 80–90% [38]. This level was also confirmed in other studies that showed that the catechin content obtained from dried gambir was about 82% [39].

**Figure 2:**
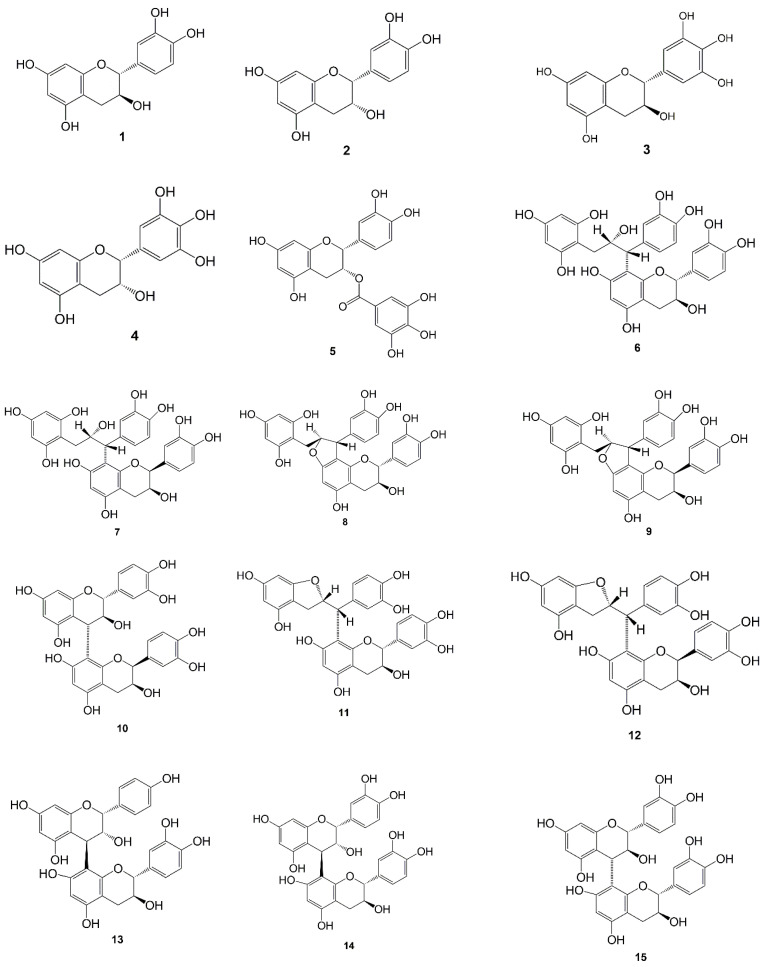

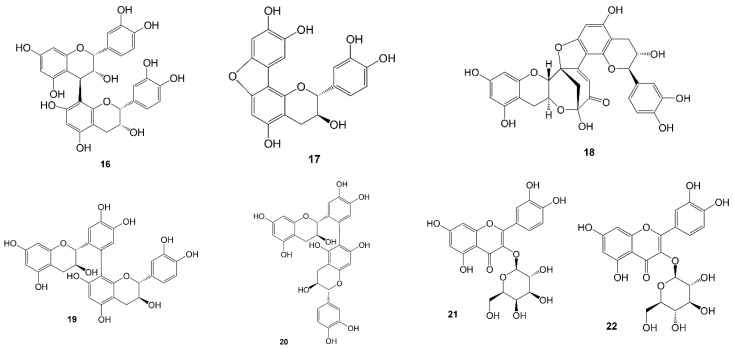
Chemical structure of flavonoid (**1**–**5**), dimeric flavonoid compounds (**6**–**20**) and flavonoid-glycosides compounds (**21**, **22**) from *Uncharia gambir* Roxb.

In another study conducted by Taniguchi et al. [40], catechins (**1**) and epicatechins (**2**) were obtained from methanol extracts, and other studies have shown that catechins can be obtained from ethanol extracts [41] and ethyl acetate extracts as well [36]. From another study, it is known that gambir contains gallocatechin (**3**), epigallocatechin (**4**), and epicatechin gallate (**5**) [36], which was isolated from ethyl acetate extract using the RP-HPLC technique. Figure 2 shows the flavonoid structure of *Uncaria gambir* Roxb.

Some of the flavonoid compounds in gambir form dimers [42]. Taniguchi et al. [40] reported four gambiriin compounds, namely, gambiriin A1 (**6**), A2 (**7**), B1 (**8**), and B2 (**9**). These four compounds have a chalcane-flavan dimer structure. This gambiriin was successfully extracted using methanol as solvent and Dia-ion HP-20 was used as a chromatographic column to fractionate the extract. Gambiriin A1 (**6**) and B2 (**9**) were obtained as amorphous powders. Meanwhile, gambiriin A2 (**7**) and B1 (**8**) were obtained as a light brown powder. However, the determined structure of gambiriins indicates that it is a product of catechins (**1**).

In addition to gambiriin A and B, other dimeric flavonoid compounds were also found in gambir. All of these flavonoid dimerics are dimeric of catechins (**1**). In 2018, Taniguchi et al. [43] found three new flavan dimers, namely, catechin-(4α→8)-ent-epicatechin (**10**), gambirflavan D1 (**11**), and gambirflavan D2 (**12**). These flavan dimers were isolated from extracts from the leaves and young twigs of gambir, and their structures were determined based on chemical and spectroscopic data. However, further research has identified other dimeric flavans namely gambiriin C (**13**), procyanidin B1 (**14**), procyanidin B3 (**15**), dimeric proanthocyanidin (**16**), gambircatechol (**17**), dehydrodicatechin A (**18**), catechin-(8→6′)-catechin (**19**), and catechin-(6→6′)-catechin (**20**). Furthermore, Yoshikado et al. [44], and Oshima et al. [45] found other flavone dimers from gambir leaves. In his research, it was explained that this dimer contained flavonoid-glycosides, namely hyperoside (**21**) and isoquercitrin (**22**) in gambir. Figure 2 shows the dimeric structure of flavonoids from *Uncaria gambir* Roxb.

**Figure 3:**
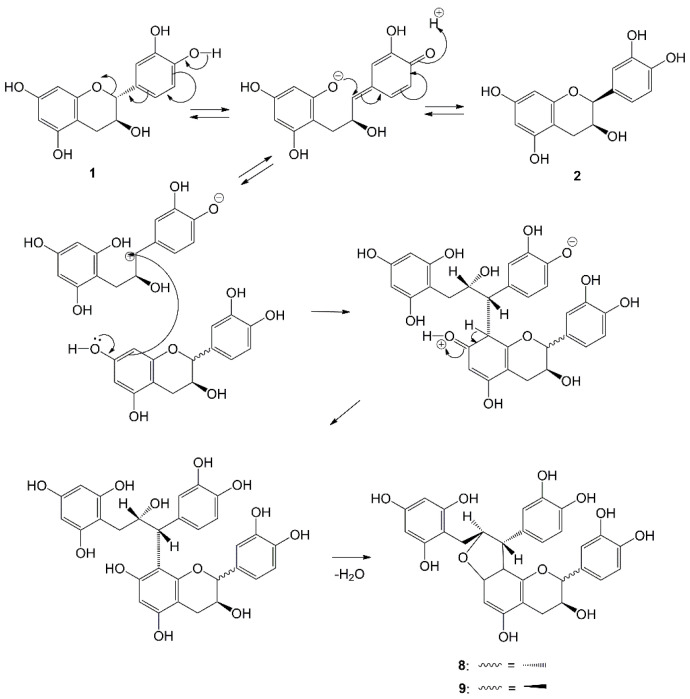
Mechanism for the Formation of Chalcane-Flavan Dimers from catechin (**1**) and or epicatechin (**2**).

The mechanism of dimer formation in gambiriin A1 (**6**) and A2 (**7**) is shown in Figure 3 [40]. The dimeric products (**6**) and (**7**) are produced by nucleophilic attack of the A-ring from catechin (**1**) (or epicatechin (**2**)) to C-α of intermediate A. Weinges et al. [46] also reported that catechins are converted to catechins, having the same planar structure as (**6**) and A2 (**7**).

**Figure 4:**
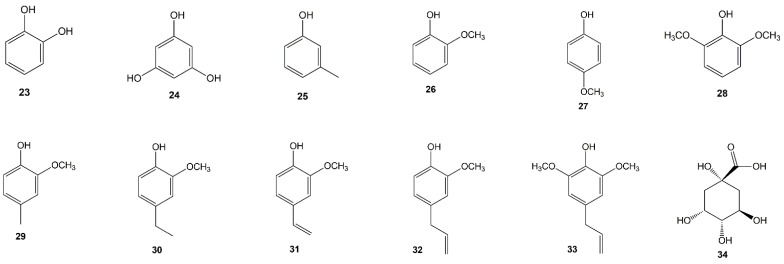
Chemical structure of free phenolic (**23**–**33**) and quinic acid (**34**) from *Uncaria gambir* Roxb.

In gambir was found free phenolic compounds such as pyrocatechol (**23**), phloroglucinol (**24**), 3-methylphenol (**25**), 2-methoxyphenol (**26**), 4-methoxyphenol (**27**), 2,6-dimethoxyphenol (**28**), 2-methoxy-4-methylphenol (**29**), 4-ethyl-2-methoxyphenol (**30**), 2-methoxy-4-vinylphenol (**31**), 4-allyl-2-methoxyphenol (**32**), 4-allyl-2,6-dimethoxyphenol (**33**), and cyclic polyol compound, namely, quinic acid (**34**). Failishnur et al. ([47] reported that pyrocatechol (**23**) could be isolated from aqueous extract that was then precipitated and analyzed using XRD. Meanwhile, phloroglucinol (**24**) was reported by Kasim et al. [48] using acetic acid extract and analyzed using GC-MS. Quinic acid (**34**) was reported by Sazwi et al. [49], showing the main compound of gambir from LC-MS/MS analysis. Figure 4 shows the free phenolic and quinic acid structure from *Uncaria gambir* Roxb.

### 3.2. Alkaloids

In 1934, the presence of an alkaloid from gambir named gambiriin was discovered, but without the provision of extraction details or chemical data. A few years later, Pavolini et al. [50] described the extraction of tannins from alkaloids with the possible formula C_22_H_26_N_2_O_4_, which they proposed under the name gambirine (**35**). Subsequently, the discovery of alkaloid compounds in gambir was reported by Merlini et al. [51]. Through chromatography, gambirtannine (**36**), dihydrogambirtannine (**37**), oxogambirtannine (**38**), and neooxygambirtannine (**39**) were identified from aqueous extracts of the leaves and stems. Afterwards, in further research, another alkaloid compound was reported, namely isogambirdine (**40**) [52,53].

**Figure 5:**
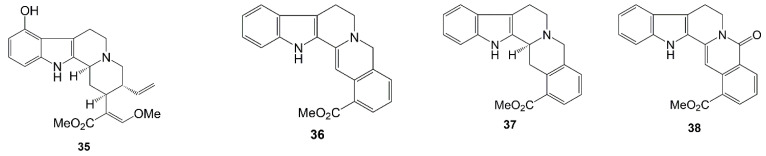

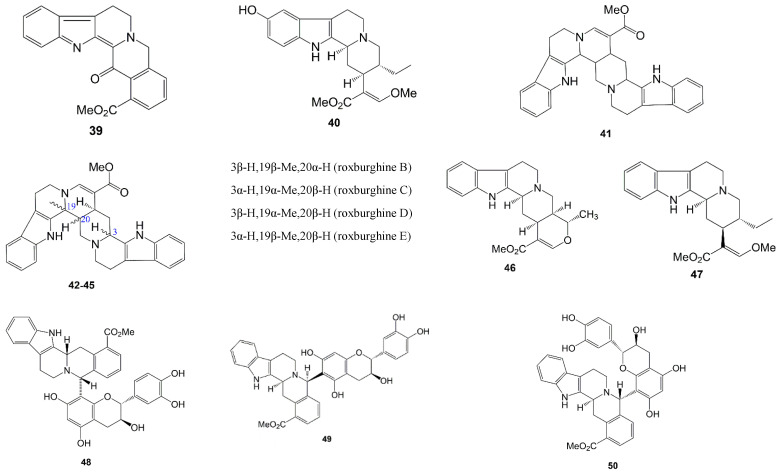
Chemical structure of alkaloid (**35**–**47**) and dimeric catechin-alkaloid compounds (**48**–**50**) from *Uncharia gambir* Roxb.

In 1970, Merlini et al. [54] were initially unaware of the presence of a new alkaloid from the methanol extract of gambir leaves. However, this extract showed a red reaction with ceric sulfate on the TLC plate indicating the presence of a base. Later on, from silica gel chromatography, five new alkaloid compounds were produced, namely, roxburghine A (**41**), B (**42**), C (**43**), D (**44**), and E (**45**). Subsequently, it was also discovered that roxburghine D (**44**) and E (**45**) were also found in the stem, along with tetrahydroalstonine (**46**) and dihydrocorynantheine (**47**). Figure 5 shows the alkaloid structure of *Uncaria gambir* Roxb.

Furthermore, Yoshikado et al. [44] reported the isolation of uncariagambiriine (**48**) from the leaves of gambir, and its unique structure is considered a catechin–alkaloid hybrid. Later, in 2019 [45], the study continued with the discovery of two compounds, namely uncariagambiriines B (**49**) and C (**50**). The structures (**49**) and (**50**), are isomers of the compound (**48**) (Figure 5).

Table 1 shows the chemical compounds from gambir. The total flavan content in the sample according to Taniguchi et al. [55] ranged from 24 to 79%. Catechins (**1**) from gambir have a lower limit of about 20% for quality management. Other flavonoids showed epicatechin (**2**) with an average of 1.5% and dimer compounds procyanidin B1 (**14**), procyanidin B3 (**15**), and gambiriin A1 (**6**) with about 1% each. Meanwhile, according to Viena & Nizar [56], the phytochemical test results of ethanol extract of gambir leaves showed the total flavonoids and total phenolic content were 32.06 and 71.80%, respectively.

### 3.3. Other Compounds

In addition to flavonoids, phenolic, and alkaloids, gambir also contains other chemical compounds such as sterols, terpenoids, saponins, carbohydrates, proteins, and amino acids [59,60]. However, there have been no studies that have comprehensively determined the structure and amount of these chemical compounds.

## 4. *Uncaria Gambir* Roxb. Uses

### 4.1. Medicinal Uses

The *Uncaria gambir* Roxb. plant has long been used in traditional medicine to treat diarrhea, sore throat, swollen gums, dysentery, arteriosclerosis, and obesity [61].

#### 4.1.1. Antioxidant

Antioxidants are compounds that can prevent damage caused by free radicals through oxidative mechanisms. Antioxidants play a role in preventing diseases such as cancer, cardiovascular disease, and premature aging [62]. Increased production of free radicals causes the growing system to require antioxidants from the outside. Antioxidants outside the body can be obtained from synthesis and naturally [63]. Synthetic antioxidants are limited in their use because they cause side effects, so natural antioxidants are needed. Gambir is a natural antioxidant [64]. The high antioxidant properties of gambir come from its content which is rich in phenolic compounds. In its utilization as an antioxidant, the most abundant phenolic components are catechins [65]. Antioxidant activity is positively correlated with the amount of catechin content [66].

Rahmawati and Fernando [67] investigated the phenolic content and antioxidant activity of the ethyl acetate extract of dried gambir leaves using the maceration method. Phenolic content using the Folin–Ciocalteu method, obtained phenolic content of 172.62 mg GAE/mL at a temperature of 80 °C, with an antioxidant activity using the DPPH method of 28.34 ppm. In addition, in the study by Sazwi et al. [49], gambir samples were extracted in distilled water for 12 h at 37 °C. The antioxidant activity using the DPPH test resulted in an IC_50_ of 6.4 ± 0.8 ppm, better than that of betel leaf and areca nut. In this study, gambir showed the presence of quinic acid as the main compound of the LC-MS/MS analysis.

However, because catechin from gambir has a high potential to be used as a natural antioxidant, extraction methods continue to be developed. One of them is the research of Widiyarti et al. [68], which has carried out the extraction of gambir leaves and twigs mechanically using a hydraulic press, screw press, and modified twin-screw press. The results of the analysis showed that the catechins extracted by mechanical means gave a catechin content of around 94.3–95.0% with an IC_50_ using the DPPH method of 4.37–4.52 ppm. In the research of Ismail et al. [69], the freeze-dryer method to produce dry extract succeeded in obtaining a total phenolic compound of 80.97 mg GAE/g with an IC_50_ of 2.74 ppm for the DPPH test, which is equivalent to the antioxidant activity of vitamin C.

#### 4.1.2. Antibacteria

**Figure 6:**
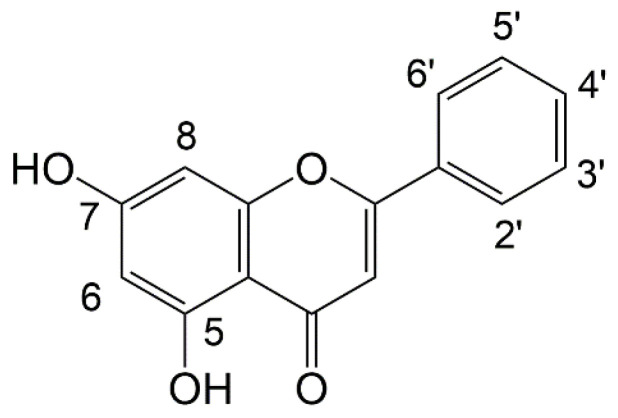
The chemical structure of flavones, it is known that hydroxylation at positions 2′,4′- or 2′,6′- on ring B and 5,7 on ring A plays an important role in antibacterial activity.

Gambir is rich in flavonoid and alkaloid compounds. Flavonoids, alkaloids, and terpenoids are known to have good activity as anti-*Streptococcus mutans*, which plays a role in dental caries [70]. Tannins including free phenolics and flavonoids have good antimicrobial activity through the inhibition of oxidative phosphorylation [71]. Flavones were also demonstrated by Tsuchiya et al. [72], who found antibacterial activity against methicillin-resistant *Staphylococcus aureus*. Those authors showed that the 2′,4′- or 2′,6′-dihydroxylated ring B and 5,7-dihydroxylated ring A in the flavanone structure were important for antibacterial activity (Figure 6).

However, the antibacterial activity of gambir extract was reported by Voravuthikunchai et al. in 2004 [73]. It is known that *Uncaria gambir* showed antimicrobial activity against *Escherichia coli* O157:H7. In this study, the aqueous extract of gambir did not have an antibacterial effect, but the ethanol extract produced an inhibition zone. Kresnawaty and Zainuddin [74] later also reported that ethanol extract has the antibacterial activity of *Escherichia coli*. In the research by Melia et al. [75], gambir was extracted with ethyl acetate as a solvent, and the antioxidant and antimicrobial activity of gambir extract were investigated. The results showed that ethyl acetate extract from gambir could inhibit the growth of *S. aureus* (Gram-positive bacteria), *E. coli*, and *Salmonella sp*. (Gram-negative bacteria). Magdalena and Kusnadi [76] tested the antibacterial activity of gambir extracted using the microwave method. The results showed that the antibacterial activity based on the diameter of the inhibition was *E. coli* ATCC 25922 of 12.07 mm, *Salmonella typhimurium* of 12.57 mm, *S. aureus* of ATCC 29213 of 13.99 mm, and *Bacillus cereus* of 14.38 mm.

To determine the main compounds that play a role in antibacterial activity, Musdja et al. [77] compared the inhibitory activity between (+)-catechins and gambir extract. The (+)-catechins were extracted from gambir using thin-layer chromatography. The antibacterial test was carried out by microdilution against *S. epidermidis*, *S. aureus*, *S. mutans*, *S. viridans*, and *Bacillus subtilis* bacteria. The results showed that catechins showed significant activity compared to the gambir extract. The minimum inhibitory concentration values for *S. epidermidis*, *S. mutans*, and *S. viridans* were 5.5; 8; and 8 mg/mL, respectively. In this study, it was also known that the antibacterial activity of (+)-catechins and gambir caused damage to plasma cell membranes and nucleoid coagulation.

#### 4.1.3. Anti-Helmintic

Helminth parasites are parasitic worms from the phyla Nematoda (roundworms) and Platyhelminthes (flatworms) [78]. Helminths are the most common infectious agents among humans in developing countries and produce diseases such as malaria and tuberculosis [79]. Between 30 and 40% of the estimated 1.4 billion people living in poverty are in sub-Saharan Africa, where helminth disease rates are also the highest [80]. Helminth disease in the human population occurs mostly in marginalized, low-income, and resource-limited areas of the world, with more than 1 billion people in developing regions of Africa, Asia, and the Americas infected with one or more types of helminths. This infection creates a burden of disease that contributes to poverty, decreased productivity, and inadequate socioeconomic development [81].

The anthelmintic potential of the ethyl acetate fraction of the alcoholic extract from the leaf and shoot of gambir showed effective anthelmintic activity against adult Indian earthworm (*Pheretima posthuma*). The extract was tested on Indian adult earthworm in vitro, and the increases in paralysis time and worm death time showed the effectiveness of gambir as a natural anthelmintic [82]. In another study, both leaf and shoot extracts were found to have anthelmintic properties in a dose-dependent manner [83]. Tannins have been reported to have anthelmintic properties because they can bind to free proteins in the digestive tract of the host resulting in their death [84,85,86].

#### 4.1.4. Anticancer

Researchers have linked the properties of gambir extract to existing diseases. Cancer is a very interesting topic because this disease is the leading cause of death worldwide, accounting for nearly 10 million deaths in 2020 [87]. Cancer is a disease in which some of the body’s cells grow uncontrollably and spread to other parts of the body. Over 100 types of cancers affect humans [88]. One of the most dangerous and common cancers is breast cancer. Breast cancer is the most common cancer in women worldwide, compared with other nonmelanoma skin cancers [89]. The incidence of breast cancer increases every year, especially in developing countries, due to increased life expectancy, lifestyle, and urbanization [90]. Previous studies have shown that the pathogenesis of breast cancer is related to the production of reactive oxygen species (ROS). Several studies have shown that plant products and plant extracts have antioxidant effects that can counteract free radicals [91].

Cancer is caused by the presence of free radicals such as superoxide anions that damage cells by forming OH, H_2_O_2_, singlet oxygen, and peroxynitrite, which can attack DNA, proteins, and fatty acids in cell membranes [92]. Gambir, which is rich in flavonoid secondary metabolite compounds, especially catechins, has antioxidant activity that can capture free radicals [93], having great potential as an anticancer. Syarifah et al. [14] investigated the anticancer activity of gambir on T47D breast cancer cells in vitro. The results showed that gambir inhibited breast cancer cell growth, although this property was still weaker than doxorubicin (DOX) as a positive control. Research on the anticancer properties of gambir extract is very limited to T47D breast cancer cells that have been reported. Meanwhile, other cancers have not been reported. Therefore, it is necessary to conduct further research on the effect of extracts from gambir on its anticancer abilities against other cells.

#### 4.1.5. Antifungal

Fungi comprise a large, heterogeneous, and ubiquitous group of heterotrophic organisms. Fungi can live as saprophytes or parasites or associate with other organisms as symbionts. Some fungi can live as parasites and can live on dead organic matter [94]. Several types of fungi play a role in the spoilage of fruits and vegetables because of their pathogenicity. Some of these fungi can produce secondary metabolites that are toxic to humans and animals [95]. Wood rot fungi are essential for the proper functioning of forest ecosystems [96]. Fungi that inhabit wood are mostly saprotrophic and utilize dead wood as food for growth and reproduction. However, some wood rot species are necrotizing parasites. Some of these fungi can attack and kill live sapwood and cause the death of live trees [97]. In addition, liver rot fungus can cause a tree to become structurally weak, and break easily due to wind [98]. The occurrence of decay in wood, as in residential construction, is responsible for enormous economic losses.

Nandika et al. [99] investigated the characteristics of catechins from gambir and their bioactivity against the wood-rot fungus *Schizophyllum commune*. Catechins were extracted from gambir through gradual maceration using hot water followed by ethyl acetate. Gambir extract was used as an antifungal because of the catechin content in gambir. Catechins are also known to show bioactivity against fungi that damage horticultural products [100]. The results showed that at concentrations of 12% or more, catechins showed remarkable bioactivity in inhibiting the growth of the wood-rotting fungus *S. commune* [99]. However, only limited research on the antifungal properties of gambir extract has been reported, and further research is needed on its effect on other fungal species.

#### 4.1.6. Anti-Inflammatory

Inflammation is a protective reaction of the microcirculation that occurs in any tissue in response to traumatic, infectious, toxic, and autoimmune injury [101,102]. Inflammation plays an important role in various diseases, such as rheumatoid arthritis, atherosclerosis, and asthma, all of which show a high prevalence globally [103]. In inflammation, free radical compounds will be produced that can cause tissue damage and trigger the biosynthesis of arachidonic acid into prostaglandins as inflammatory mediators [104].

The anti-inflammatory activity of two extracts from the leaf of *Uncaria gambir* and root bark of *Morus alba* (1:1) was observed in vivo by Yimam et al. [105]. The results showed that the highest dose of 300 mg reduced inflammation by 53.7%, 55.3%, and 48.8%, observed at 1 h, 3 h, and 5 h. In addition, in this study, the extract mixture was tested in vitro for the inhibitory activity of cyclooxygenase-2, COX-2, and lipoxygenase enzymes. The results showed that the extracts had IC_50_ values for cyclooxygenase 2, COX 2, and lipoxygenase enzyme activity of 12.4; 39.8; and 13.6 g/mL, respectively.

In 2019, Musdja et al. [106] studied in vivo catechins isolated from gambir using ethyl acetate solvent with Winter’s modified paw edema method. This study reported that the best dose for inhibiting edema was 100 mg/kg BW with a concentration of 59.19%. Similar results were also reported by Yunarto et al. [107], who used 25 white rats of the Wistar strain as an animal assay that dose 5, 10, and 20 mg/kg BW. The results showed that the ethyl acetate fraction of *Uncaria gambir* leaves at all doses had an anti-inflammatory effect.

#### 4.1.7. Anti-Hyperglycemic

Diabetes or diabetes mellitus (DM) is a metabolic disorder characterized by high blood glucose levels called hyperglycemia [108,109]. Metabolic disorders caused by pancreatic cell damage result in decreased insulin secretion resulting in hyperglycemia as a symptom of diabetes that causes complications in body parts and increases the risk of premature death [110]. One therapeutic approach to reducing hyperglycemia is to slow down glucose absorption by inhibiting carbohydrate hydrolysis enzymes, such as α-amylase, α-glucosidase, sucrose, and maltose [15,111].

Several studies have been conducted to determine the effect of gambir extract on reducing blood sugar levels [112,113]. Widiyarti et al. [114] have isolated catechins by extraction method using ethyl acetate as an anti-hyperglycemic agent in vitro. This anti-hyperglycemic activity was analyzed as a α-glucosidase inhibitor. The results showed that the IC_50_ for α-glucosidase inhibition ranged from 40.45 to 52.43 g/mL, so it could be classified as a good antidiabetic. Meanwhile, Apea-Bah et al. [115] showed that ethanolic and ethyl acetate extracts had significantly higher α-glucosidase inhibitory activity than aqueous extracts, with IC_50_ ranges of 15.2–49.5 g/mL. Apart from α-glucosidase inhibitors, Viena and Nizar (2018) investigated antidiabetic properties through α-amylase enzyme inhibition. The results showed that the ethanol extract of gambir leaves had α-amylase inhibitory activity of 88.22% at a sample concentration of 1000 ppm.

Meanwhile, in the research of Zebua et al. [16], in vivo anti-hypoglycemic activity with gambir extract using distilled water, ethyl acetate, and ethanol solvents was tested on alloxan-induced mice. The results showed that extraction using distilled water produced the best activity. Aqueous extract from gambir at a dose of 300 mg/kg BW given for 15 days reduced blood glucose levels by 50.62%, and increased body weight by 10.18%. In summary, gambir extract is a potential anti-hyperglycemic agent. In addition, molecular studies need to be carried out to determine the mechanism of action of its bioactive compounds as anti-hyperglycemic agents.

#### 4.1.8. Anti-Hyperuricemia

Hyperuricemia is a metabolic disorder condition characterized by high uric acid levels due to xanthine oxidase (XO) activity [115,116,117]. Rismana et al. [41] investigated gambir as an inhibitor of the xanthine oxidase enzyme. Ethanol extract from gambir leaf that was used as an XO inhibitor in vitro showed high activity of 50% relative to standard allopurinol at 100 ppm of final concentration.

#### 4.1.9. Anti-Lipid Peroxidation Activity

The increased formation of reactive oxygen species (ROS) causes tissue dysfunction and damage in a number of pathological conditions. ROS oxidize lipids to produce peroxides and aldehydes. These lipid peroxidation products are much more stable than the parent ROS and can diffuse. Therefore, lipid oxidation products can expand and propagate response including injury. Lipid peroxidation products (LPOs) are highly reactive and cause selective changes in cell signaling, protein and DNA damage, and cytotoxicity [118,119]. In 2019, Ningsih et al. [120] studied ethanol extract from gambir via anti-lipid peroxidation by in vitro. Ethanol extract of gambir was used for the inhibition of lipid peroxidation in rat liver homogenates. The results showed the highest activity for 96% ethanol extract with an IC_50_ of 24.6 ppm, which was equivalent to a positive control of polyphenon 60. HPLC analysis also showed that the extract contained 98% phenolic compounds of which 59% were (+)-catechins.

#### 4.1.10. Antihyperlipidemic and Atherosclerosis

Hyperlipidemia is a major risk factor in atherosclerosis. Meanwhile, atherosclerosis is the accumulation of fat in the tunica intima matrix, followed by the formation of connective tissue in the walls of blood vessels [121]. Atherosclerosis is a risk factor for coronary heart disease [122,123]. Several studies have shown that gambir can be used as an antihyperlipidemic [124,125]. Yunarto et al. [126] used ethyl acetate extract from gambir leaves as an antihyperlipidemic in vivo. The authors used 36 male white rats of the Sprague–Dawley strain, 2.5 months old and induced with food containing saturated fat and cholesterol for 28 days. The results showed that a dose of 20 mg/200 g BW was able to increase HDL levels and reduce total triglycerides, LDL, and cholesterol.

In 2019, Alioes et al. [127] tested catechin isolates from gambir extract against triacylglycerol levels in vivo. Triacylglycerol is a compound consisting of three fatty acids and glycerol linked through ester bonds [128] and has an important role in the transport and storage of lipids [129]. This study tested catechin isolates from gambir extract against triacylglycerol levels in rats. The study was conducted with 25 rats given a diet high in beef brain fat for 14 days. Triacylglycerol levels were analyzed using the glycerophosphate oxidase method. The results showed a decrease in triacylglycerol levels after catechin isolate was given, with the highest decrease at a dose of 40 mg/kg body weight/day, which was 110 ± 3.2 mg/dL.

### 4.2. Non-Medicinal Uses

#### 4.2.1. Adsorbent of Dye

**Figure 7:**
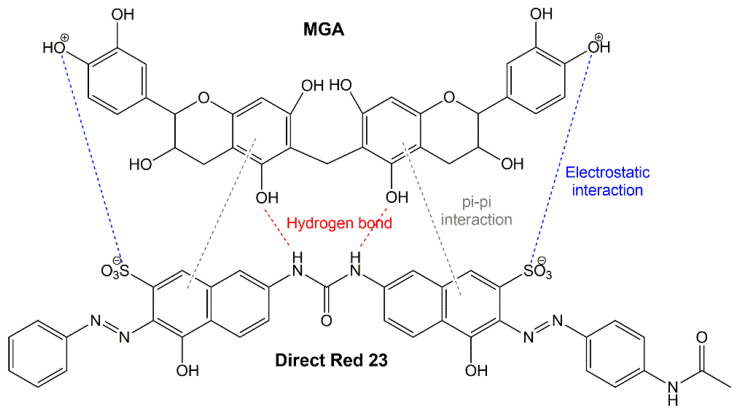
Mechanism of direct red 23 adsorption to modified gambir adsorbent (MGA).

In addition to biological activity, gambir extract was chemically modified for developing novel green adsorbents. Achmad et al. [130] reported that modified gambir adsorbent (MGA) adsorbed direct red dye 23. Gambir was defatted with n-hexane and extracted with ethyl acetate solvent. Then, the solvent was evaporated to obtain the powder. Gambir extract was modified by added formaldehyde and HCl. The experimental result shows that the adsorption mechanism was well described by the Langmuir isotherm model, with an adsorption capacity of 26.67 mg/g. The mechanism for the adsorption of direct red 23 to MGA is shown in Figure 7. The interactions that occur between MGA and direct red 23 are electrostatic interactions, π-π interactions, and hydrogen bonds.

In addition, in the research by Tong et al. [131], gambir was chemically modified into an insoluble adsorbent to become an adsorbent of Cu^2+^ ions. Gambir extracted with 70% aqueous acetone, then added formaldehyde as a cross-linking agent. In the results, the adsorbent exhibited equilibrium data that matched the pseudo-second-order equation in which the adsorption process is chemisorptions. The maximum adsorption capacity was found to be 9.95 mg/g at 333 K, indicating that the process of Cu^2+^ ions being adsorbed onto the gambir took place spontaneously with a negative Gibbs free energy change.

However, the use of gambir extract as an adsorbent has high potential because it contains compounds that have many functional groups such as hydroxy, carbonyl, and aromatic that have π bonds. The large number of functional groups will encourage bonds between extract compounds from gambir and liquid waste, thereby producing good adsorption abilities.

#### 4.2.2. Corrosion Inhibition

Research by Hussin and Kassim [132] showed that ethyl acetate extract from gambir had an inhibitory effect on mild steel corrosion in 1 M HCl solution. The catechins contained in the extract could reduce the corrosion rate of mild steel in acid solution. The results showed that this extract behaved as a mixed-type corrosion inhibitor with the highest inhibition at 1000 ppm. In addition, the surface analysis showed that there were some improvements in the surface morphology after the addition of gambir extract inhibitor. This extract had a percentage inhibition efficiency of 97.25%. This result is higher than the research conducted by Ogunleye et al. [133], who used *Luffa cylindrica* leaf extract, which has an inhibition efficiency of 87.89%.

## 5. Conclusions

*Uncaria gambir* Roxb. has spread in Southeast Asia, especially Indonesia and Malaysia. *U. gambir* Roxb. has many traditional uses, namely in the treatment of wounds, ulcers, asthma, headaches, bacterial/fungal infections, tooth pain, cancer, cirrhosis, diabetes, rheumatism, dysentery, and urinary tract inflammation. In addition, 50 compounds were detected in the leaves, twigs, wood, bark, and stems of *U. gambir*. These compounds are flavonoids, free aromatics, alkaloids, glucose, dimeric compounds from these groups, and so forth. The main bioactive compounds extracted from *U. gambir* are catechins. Catechins are major compounds and determine the pharmacological activity in this plant extract.

Extracts provide various bioactivities such as antioxidant, antibacterial, anti-helmintic, anticancer, antifungal, anti-inflammatory, anti-hyperglycemic, anti-hyperuricemic, anti-lipid peroxidation, and antihyperlipidemic. Therefore, these various bioactivities prove the potential of this plant to be applied in treating many diseases. In addition, *U. gambir* Roxb. showed other activities as adsorbent of dye and corrosion inhibition. However, further studies to determine the mechanism, adsorption ability for other dyestuffs, and the role of the components in the extract have not been investigated. In conclusion, further intensive investigations are recommended to explore the molecular mechanism of each component of the extract for its medical uses, and also to investigate more applications of the non-medical uses of *U. gambir* Roxb.

## Figures and Tables

**Table 1:** Compounds identified from *Uncaria gambir* Roxb.

No	Compound	Type	Extract	Plant Part	Ref.
**1**	Catechin	Flavonoid	Water	Leaves	[37]
			Water	Leaves and young twigs	[47,57]
			Methanol	Leaves and young twigs	[40]
			Ethanol	Leaves	[41]
			Ethyl acetate	Leaves and young twigs	[36]
**2**	Epicatechin	Flavonoid	Water	Leaves	[37]
			Methanol	Leaves and young twigs	[40]
			Ethyl acetate	Leaves and young twigs	[36]
**3**	Gallocatechin	Flavonoid	Ethyl acetate	Leaves and young twigs	[36]
**4**	Epigallocatechin	Flavonoid	Ethyl acetate	Leaves and young twigs	[36]
**5**	Epicatechin gallate	Flavonoid	Ethyl acetate	Leaves and young twigs	[36]
**6**	Gambiriin A1	Dimeric flavonoid	Methanol	Leaves and young twigs	[40]
**7**	Gambiriin A2	Dimeric flavonoid	Methanol	Leaves and young twigs	[40]
**8**	Gambiriin B1	Dimeric flavonoid	Methanol	Leaves and young twigs	[40]
**9**	Gambiriin B2	Dimeric flavonoid	Methanol	Leaves and young twigs	[40]
**10**	Catechin-(4α→8)-ent-epicatechin	Flavonoid	Methanol	Leaves and young twigs	[43]
**11**	Gambiriin C	Dimeric flavonoid	Methanol	Leaves and young twigs	[40]
**12**	Procyanidin B1	Dimeric flavonoid	Methanol	Leaves and young twigs	[40]
**13**	Procyanidin B3	Dimeric flavonoid	Methanol	Leaves and young twigs	[40]
**14**	Dimeric proanthocyanidin	Dimeric flavonoid	Methanol	Leaves and young twigs	[40]
**15**	Gambirflavan D1	Dimeric flavonoid	Methanol	Leaves and young twigs	[43]
**16**	Gambirflavan D2	Dimeric flavonoid	Methanol	Leaves and young twigs	[43]
**17**	Gambircatechol	Dimeric flavonoid	Acetone	Leaves	[44]
**18**	Dehydrodicatechin A	Dimeric flavonoid	Acetone	Leaves	[44]
**19**	Catechin-(8→6′)-catechin	Dimeric flavonoid	Acetone	Leaves	[44]
**20**	Catechin-(6→6′)-catechin	Dimeric flavonoid	Acetone	Leaves	[44]
**21**	Hyperoside	Flavonoid-glycosides	Water	Leaves and twigs	[45]
**22**	Isoquercitrin	Flavonoid-glycosides	Water	Leaves and twigs	[45]
**23**	Pyrocatechol	Phenolic	Water	Leaves	[37]
			Water	Leaves and young twigs	[47]
			Acetone	Leaves and young twigs	[48]
**24**	Phloroglucinol	Phenolic	Acetic acid	Leaves and young twigs	[48]
**25**	3-methylphenol	Phenolic	Methanol	Wood	[58]
**26**	2-methoxyphenol	Phenolic	Methanol	Wood and bark	[58]
**27**	4-methoxyphenol	Phenolic	Methanol	Wood and bark	[58]
**28**	2,6-dimethoxyphenol	Phenolic	Methanol	Wood and bark	[58]
**29**	2-methoxy-4-methylphenol	Phenolic	Methanol	Wood and bark	[58]
**30**	4-ethyl-2-methoxyphenol	Phenolic	Methanol	Wood and bark	[58]
**31**	2-methoxy-4-vinylphenol	Phenolic	Methanol	Wood and bark	[58]
**32**	4-allyl-2-methoxyphenol	Phenolic	Methanol	Wood and bark	[58]
**33**	4-allyl-2,6-dimethoxyphenol	Phenolic	Methanol	Wood and bark	[58]
**34**	Quinic acid	Cyclic polyol	Water	All part	[49]
**35**	Gambirine	Alkaloid	Methanol	Leaves	[50]
**36**	Gambirtannine	Alkaloid	Methanol	Leaves	[51]
**37**	Dihydrogambirtannine	Alkaloid	Methanol	Leaves	[51]
**38**	Oogambirtannine	Alkaloid	Methanol	Leaves	[51]
**39**	Neooxygambirtannine	Alkaloid	Methanol	Leaves	[51]
**40**	Isogambirine	Alkaloid	Methanol	Leaves	[52]
**41**	Roxburghines A	Alkaloid	Methanol	Leaves	[54]
**42**	Roxburghines B	Alkaloid	Methanol	Leaves	[54]
**43**	Roxburghines C	Alkaloid	Methanol	Leaves	[54]
**44**	Roxburghines D	Alkaloid	Methanol	Leaves and stems	[54]
**45**	Roxburghines E	Alkaloid	Methanol	Leaves and stems	[54]
**46**	Tetrahydroalstonine	Alkaloid	Methanol	Leaves and stems	[54]
**47**	Dihydrocorynantheine	Alkaloid	Methanol	Leaves and stems	[54]
**48**	Uncariagambiriine	Catechin-alkaloid	Acetone	Leaves	[44]
**49**	Uncariagambiriine B	Catechin-alkaloid	Water	Leaves and twigs	[45]
**50**	Uncariagambiriine C	Catechin-alkaloid	Water	Leaves and twigs	[45]

## Data Availability

The study not report any data.

## Sample Availability

Samples of the compounds are not available from the authors.
